# Antimicrobial Resistance, Biofilm Formation, and Phylogenetic Distribution of *Escherichia coli* in Hospitalized Patients with Community-Onset Urinary Tract Infections in Western Mexico

**DOI:** 10.3390/antibiotics15060541

**Published:** 2026-05-27

**Authors:** Luis Asdrúval Zepeda-Gutiérrez, Sol Ramírez-Ochoa, Mauricio Alfredo Ambriz-Alarcón, Enrique Cervantes-Pérez, Araceli Castillo-Romero, Karel Cesar Licona-Lasteros, Rafael Cortés-Zárate

**Affiliations:** 1 Programa de Maestría en Microbiología Médica, Centro Universitario de Ciencias de la Salud, Universidad de Guadalajara, Guadalajara 44340, Jalisco, Mexico; luis.zepeda5322@alumnos.udg.mx; 2 Departamento de Medicina Interna, Hospital Civil de Guadalajara Fray Antonio Alcalde, Universidad de Guadalajara, Guadalajara 44280, Jalisco, Mexico; ramirez_ochoa_sol@hotmail.com (S.R.-O.); mau_ambriz@hotmail.com (M.A.A.-A.); enrique.cervantes@academico.udg.mx (E.C.-P.); 3 Centro de Investigación de Enfermedades Infectocontagiosas, Departamento de Microbiología y Patología, Centro Universitario de Ciencias de la Salud, Universidad de Guadalajara, Sierra Mojada 950, Col. Independencia, Guadalajara 44340, Jalisco, Mexico; araceli.castillo@academicos.udg.mx; 4 Laboratorio de Sistemas Biológicos, Departamento de Ciencias de la Salud, Centro Universitario de los Valles, Universidad de Guadalajara, Carretera Guadalajara—Ameca Km. 45.5, Ameca 46600, Jalisco, Mexico

**Keywords:** *Escherichia coli*, urinary tract infection, antimicrobial resistance, ESBL, multidrug resistance, biofilm formation, phylogenetic groups

## Abstract

Background/Objectives: *Escherichia coli* is the predominant pathogen in community-onset urinary tract infections (UTIs) requiring hospitalization. This study characterized antimicrobial resistance profiles, biofilm formation, extended-spectrum β-lactamase (ESBL) gene distribution, and phylogenetic background of *E. coli* isolates from hospitalized UTI patients in Western Mexico. Methods: Seventy isolates (September 2023–September 2024) underwent susceptibility testing (CLSI M100, 35th edition), multiplex PCR for *bla*TEM, *bla*CTX-M, and *bla*SHV genes, crystal violet biofilm quantification, and Clermont quadruplex PCR phylotyping. Associations were evaluated by Fisher’s exact test with Benjamini–Hochberg FDR (BH-FDR) correction. Results: ESBL phenotype and MDR were detected in 57.1% and 58.6% of isolates. After BH-FDR correction, ESBL production was significantly associated with amikacin (OR = 5.55; 95% CI: 1.80–18.74; q = 0.002) and TMP-SMX non-susceptibility (OR = 3.00; 95% CI: 1.02–9.23; q = 0.036); ciprofloxacin non-susceptibility was linked to MDR status (OR = 7.21; 95% CI: 1.28–75.66; q = 0.017) but not ESBL phenotype. Biofilm was detected in 77.1% of isolates. *bla*TEM predominated among ESBL producers (85.0%). Phylogroup B2 (51.4%) was inversely associated with recurrent UTI on both univariate (OR = 0.17; 95% CI: 0.03–0.73; *p* = 0.008) and adjusted analysis (adjusted OR = 0.19; 95% CI: 0.05–0.81; *p* = 0.025). Phylogroup C (22.9%) exhibited the highest MDR prevalence (81.3%) and the highest biofilm formation rate among phylogroups (87.5%). Conclusions: The high prevalence of ESBL-producing and MDR *E. coli*, combined with an unexpected predominance of *bla*TEM, reveals a distinctive local resistance landscape diverging from regional trends. The inverse association of phylogroup B2 with recurrence and TMP-SMX resistance reinforces the clinical value of phylogenetic surveillance in guiding UTI management strategies.

## 1. Introduction

Urinary tract infections (UTIs) are among the most common bacterial infectious diseases in humans and represent a major global public health problem [1], affecting more than 150 million people each year, with an estimated economic impact exceeding 6 billion dollars annually. Women are the most affected, with a 50–60% probability of experiencing at least one episode of a UTI in their lifetime. Uropathogenic *Escherichia coli* (UPEC) strains are responsible for approximately 80–90% of community-acquired UTIs and a significant proportion of complicated (including pyelonephritis and sepsis) or hospital-acquired infections [2].

Patients may require inpatient monitoring and hospital-based antimicrobial therapy for several reasons: severe clinical manifestations such as pyelonephritis or septic shock [3], persistent or recurrent presentations frequently associated with biofilm formation [4], and failures of outpatient treatment attributable to antimicrobial resistance [1].

In Mexico, a multidrug resistance rate of 78% has been reported in UPEC strains, significantly reducing the available therapeutic options, with high levels of resistance to trimethoprim-sulfamethoxazole (62%), fluoroquinolones (57%), and β-lactam antibiotics (80%), including third-generation cephalosporins (57%). This last group is mainly explained by the dissemination of extended-spectrum beta-lactamases (ESBLs). The most frequently reported ESBLs are CTX-M-1 (56%), followed by TEM-1 (45%) and CTX-M-15 (41%) [5,6,7]. Some studies in Mexico have associated antimicrobial resistance with biofilm formation, particularly resistance to second- to fourth-generation cephalosporins [8]. This is particularly relevant since a prevalence of biofilm-forming UPEC ranging from 74% to 100% has been reported [6,8,9].

Certain *E. coli* phylogroups, particularly those belonging to groups B2 and D, are associated with urinary tract infections due to their higher virulence potential [10]. These strains typically carry multiple factors that promote colonization and invasion of the urinary tract, conferring a greater ability to cause complicated, recurrent, and systemic infections [11].

Despite growing evidence on antimicrobial resistance and virulence factors in UPEC, the interplay between resistance profiles, biofilm formation, and phylogenetic background in hospitalized patients remains insufficiently characterized, particularly in Latin American settings [12]. Therefore, this study aimed to determine the association between antimicrobial resistance patterns, biofilm-forming capacity, and phylogenetic groups of *Escherichia coli* strains isolated from adult patients who required hospitalization due to urinary tract infection.

## 2. Results

Seventy *E. coli* isolates were recovered from patients meeting all inclusion criteria. The mean age was 53.3 years (±19.6), with women comprising the majority (67.1%). The most prevalent comorbidities were type 2 diabetes mellitus (30.0%) and a history of renal transplantation (18.6%), reflecting the referral profile of this tertiary care center. Pyelonephritis was the predominant clinical presentation (51.4%), and UTI-attributable complications occurred in 27.1% of patients, with septic shock being the most frequent (52.6% of complicated cases). Four patients (5.7%) died due to infection severity. Full demographic and clinical data are presented in Table 1.

### 2.1. Antimicrobial Resistance

Antimicrobial susceptibility testing results are detailed in Table 2. Using strict resistance (R) criteria, the highest resistance rates were observed for ciprofloxacin (78.6%; 95% CI: 67.1–87.5), trimethoprim-sulfamethoxazole (TMP-SMX; 58.6%; 95% CI: 46.2–70.2), cefuroxime (58.6%; 95% CI: 46.2–70.2), cefotaxime (57.1%; 95% CI: 44.7–68.9), ceftazidime (55.7%; 95% CI: 43.3–67.6), and aztreonam (55.7%; 95% CI: 43.3–67.6).

When non-susceptibility (NS; combining R, Intermediate, and Susceptibility Dose-Dependent categories) was applied as the outcome measure in accordance with WHO-GLASS epidemiological criteria, resistance burden increased substantially for several agents. Notably, ciprofloxacin NS reached 85.7% (95% CI: 75.3–92.9), amikacin increased from 27.1% (R only) to 50.0% (95% CI: 37.8–62.2), amoxicillin-clavulanate from 27.1% to 48.6% (95% CI: 36.4–60.8), cefepime from 34.3% to 52.9% (95% CI: 40.6–64.9)—driven by 13 isolates (18.6%) classified as susceptibility dose-dependent—and piperacillin-tazobactam from 30.0% to 42.9% (95% CI: 31.1–55.3).

In contrast, fosfomycin and nitrofurantoin maintained favorable susceptibility profiles, with NS rates of 4.3% (95% CI: 0.9–12.0) and 11.4% (95% CI: 5.1–21.3), respectively. Complete susceptibility (0% resistance) was observed for meropenem and cefoxitin across all 70 isolates, confirming the absence of carbapenem resistance in this collection.

Overall, 40 isolates (57.1%) exhibited the ESBL phenotype, and 41 isolates (58.6%) met the criteria for multidrug resistance (MDR), defined as non-susceptibility to at least one agent in three or more antimicrobial categories, in accordance with the international consensus definitions proposed by Magiorakos et al. [13]. No isolate met the criteria for extensive drug resistance (XDR) or pan-drug resistance (PDR), as all isolates retained susceptibility to at least three antimicrobial categories, including carbapenems and cephamycins.

Non-susceptibility rates according to ESBL-producing and MDR phenotypes are presented in Table 3. ESBL-producing isolates exhibited significantly higher non-susceptibility to all β-lactam agents tested, including cephalosporins (2nd through 4th generation), aztreonam, and piperacillin-tazobactam (OR = 30.74; 95% CI: 6.20–304.89; q < 0.001), as well as amoxicillin-clavulanate (OR = 28.94; 95% CI: 6.76–183.76; q < 0.001), after BH-FDR correction (Table 3).

Among non-β-lactam agents, ESBL producers showed significantly higher non-susceptibility to amikacin (67.5% vs. 26.7%; OR = 5.55; 95% CI: 1.80–18.74; q = 0.002), trimethoprim-sulfamethoxazole (70% vs. 43.3%; OR = 3.00; 95% CI: 1.02–9.23; q = 0.036), and nitrofurantoin (20% vs. 0%; q = 0.012). Ciprofloxacin non-susceptibility did not reach significance for the ESBL phenotype (92.5% vs. 76.7%; q = 0.094).

A broadly similar pattern was observed for MDR isolates, with significant non-susceptibility across all β-lactam classes (q < 0.001 for all) and amikacin (68.3% vs. 24.1%; OR = 6.56; 95% CI: 2.07–23.24; q < 0.001) and nitrofurantoin (19.5% vs. 0%; q = 0.021). However, two phenotype-specific associations were identified: ciprofloxacin non-susceptibility was significantly associated with MDR status (95.1% vs. 72.4%; OR = 7.21; 95% CI: 1.28–75.66; q = 0.017) but not with ESBL production, while trimethoprim-sulfamethoxazole showed the inverse pattern, reaching significance only for the ESBL phenotype. Fosfomycin non-susceptibility was not significantly associated with either phenotype (q > 0.25 for both). Meropenem and cefoxitin showed complete susceptibility across all isolates regardless of phenotype.

To assess whether the ESBL and MDR associations identified above persisted after adjustment for potential clinical confounders, binary logistic regression models were constructed (Supplementary Tables S2 and S5). For ESBL production, no clinical covariate reached statistical significance after adjustment; however, renal transplant history (adjusted OR = 4.35; 95% CI: 0.93–20.45; *p* = 0.063) and type 2 diabetes mellitus (adjusted OR = 3.44; 95% CI: 0.98–11.99; *p* = 0.053) showed borderline associations. These results indicate that the ESBL phenotype in this collection was not independently determined by the demographic and clinical factors available, consistent with a community-driven ecological dynamic rather than host-specific selection. For MDR status, both renal transplant history (adjusted OR = 6.29; 95% CI: 1.12–35.28; *p* = 0.037) and type 2 diabetes mellitus (adjusted OR = 4.06; 95% CI: 1.15–14.40; *p* = 0.030) remained statistically significant after adjustment for age and sex, identifying these comorbidities as independent clinical correlates of MDR phenotype. All models showed adequate fit (Hosmer–Lemeshow *p* > 0.05 for all) and no evidence of multicollinearity (maximum VIF = 1.41).

### 2.2. Genotyping of ESBL-Producing Strains

**Figure 1:**
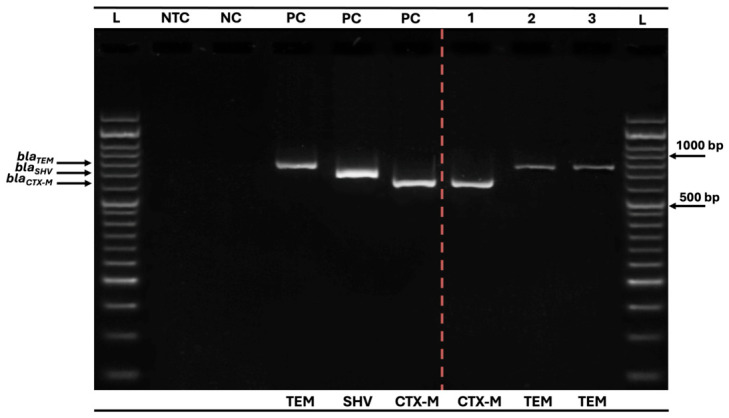
Multiplex PCR for the detection of extended-spectrum β-lactamases (*bla* genes). From left to right: L, DNA molecular weight marker; NTC, No Template Control (reaction mixture without DNA template); NC, negative control (*E. coli* ATCC 25922); PC (TEM), positive control (*E. coli* ATCC 35218 producing *bla*TEM-1); PC (SHV), positive control (*Klebsiella pneumoniae* ATCC 700603 producing *bla*SHV-18); PC (CTX-M), positive control (*E. coli* strain producing *bla*CTX-M). To the right of the red dashed line: lane 1, clinical strain identified as *bla*CTX-M producer; lane 2, clinical strain identified as *bla*TEM producer; lane 3, clinical strain identified as *bla*TEM producer. Electrophoresis was performed on a 3% agarose gel in 1× TBE buffer at 80 V for 90 min. L, DNA ladder (shown on both sides of the gel); NTC, No Template Control; NC, negative control; PC, positive control.

PCR electrophoresis products were visualized for all 40 isolates (57.1%) that met the ESBL phenotype criteria (Figure 1). Among the 40 ESBL-producing isolates, a single *bla* gene was detected in 34 (85%), comprising *bla*TEM alone in 28 (70.0%), *bla*CTX-M alone in 4 (10.0%), and *bla*SHV alone in 2 (5.0%). Gene co-carriage was identified in 6 isolates (15%): *bla*TEM/*bla*CTX-M in 3 (7.5%) and *bla*TEM/*bla*SHV in 3 (7.5%). No isolate harbored all three genes simultaneously. No significant associations were found between the different β-lactamase genes and clinical variables.

### 2.3. Biofilm Formation

Based on the absorbance values and the calculated cutoff optical density, biofilm production was classified by intensity according to the established criteria [14]. Biofilm formation was observed in 54 of 70 isolates (77.1%), with weak, moderate, and strong production in 42 (60.0%), 8 (11.4%), and 4 (5.7%) isolates, respectively. No statistically significant differences were identified across biofilm intensity categories with respect to ESBL phenotype (*p* = 0.060), MDR status (*p* = 0.304), resistance gene carriage (*bla*TEM *p* = 0.155; *bla*CTX-M *p* = 0.083; *bla*SHV *p* = 0.230), or clinical outcomes, including complication rate (*p* = 0.854) and mortality (*p* = 0.234). Urinary catheter use was numerically more frequent among weak biofilm producers (16.7%) compared with the remaining intensity categories, consistent with the role of indwelling devices in early biofilm initiation; however, this difference did not reach statistical significance (*p* = 0.263). Full stratified data are provided in Supplementary Table S1. These findings support the independence of biofilm-forming capacity from antimicrobial resistance mechanisms in this collection, consistent with previously reported evidence that biofilm regulation is governed primarily by virulence-associated pathways rather than by horizontally acquired resistance determinants.

### 2.4. Phylogroups

**Figure 2:**
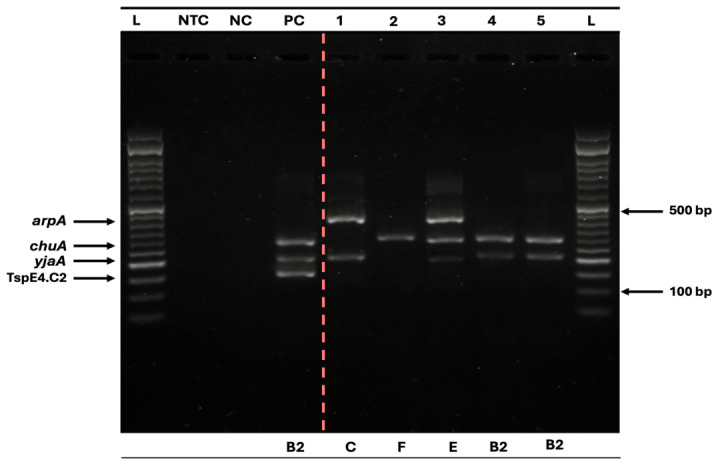
Quadruplex PCR for *Escherichia coli* phylogenetic group determination using the Clermont typing method [15]. From left to right: L, DNA molecular weight marker; NTC, No Template Control (reaction mixture without DNA template); NC, negative control (*Klebsiella pneumoniae* ATCC 700603); PC, positive control (*E. coli* ATCC 25922, phylogroup B2). To the right of the red dashed line: lane 1, clinical strain typed as phylogroup C; lane 2, clinical strain typed as phylogroup F; lane 3, clinical strain typed as phylogroup E; lanes 4 and 5, clinical strains typed as phylogroup B2; L, DNA molecular weight marker. Electrophoresis was performed on a 2% agarose gel in 1× TBE buffer at 80 V for 100 min. L, DNA ladder (shown on both sides of the gel); NTC, No Template Control; NC, negative control; PC, positive control.

The 70 *E. coli* isolates were phylogenetically classified using PCR product electrophoresis (Figure 2).

**Figure 3:**
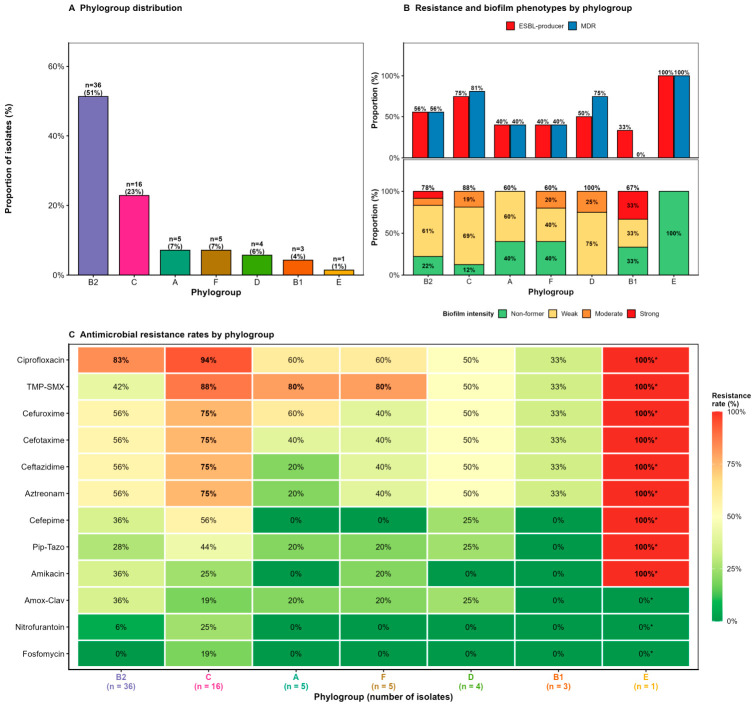
Integrative overview of phylogenetic distribution, resistance phenotypes, and antimicrobial resistance rates in *Escherichia coli* isolates from hospitalized patients with community-onset urinary tract infections in Western Mexico (*N* = 70). (**A**) Phylogroup distribution; isolate counts (*n*) and proportions (%) are shown for each phylogroup. (**B**, **upper panel**) Prevalence of ESBL-producing and MDR phenotypes per phylogroup; these proportions are not directly readable from Table 4 due to the cross-tabulated format. (**B**, **lower panel**) Biofilm intensity distribution per phylogroup; labels above bars represent the total percentage of biofilm-forming isolates (Weak + Moderate + Strong combined). (**C**) Heatmap of antimicrobial non-susceptibility rates (% non-susceptible isolates, NS = R + I + SDD) stratified by phylogroup, enabling simultaneous visual identification of resistance gradients across 12 agents and 7 phylogroups—a pattern not discernible from tabular presentation. Color gradient: green = low non-susceptibility, red = high non-susceptibility. Bold values indicate ≥75% non-susceptibility. Asterisk (*) denotes phylogroups with *n* < 5 isolates; all results for these groups are purely descriptive and should not be used for inferential purposes. Cefoxitin and meropenem (100% susceptibility across all isolates) were excluded. ESBL, extended-spectrum β-lactamase; MDR, non-susceptibility to ≥1 agent in ≥3 antimicrobial categories; TMP-SMX, trimethoprim–sulfamethoxazole; Pip-Tazo, piperacillin–tazobactam; Amox-Clav, amoxicillin–clavulanate.

Phylogenetic classification of the 70 *E. coli* isolates revealed a predominance of phylogroup B2 (36/70; 51.4%), followed by phylogroups C (16/70; 22.9%), A and F (5/70 each; 7.1%), D (4/70; 5.7%), B1 (3/70; 4.3%), and E (1/70; 1.4%) (Figure 3A). Phenotypic and clinical characteristics stratified by phylogroup are presented in Table 4. Phylogroups with fewer than five isolates (D, B1, and E) are reported descriptively only; statistical comparisons involving these groups should be interpreted with caution given the limited sample size.

The prevalence of ESBL-producing strains was highest in phylogroup E (1/1; 100%), followed by C (12/16; 75%) and B2 (20/36; 55.6%). MDR phenotype was most frequent in phylogroup C (13/16; 81.3%) and D (3/4; 75.0%) (Figure 3B, upper panel). Biofilm formation was universal in phylogroup D (4/4; 100%) and most prevalent in C (14/16; 87.5%) and B2 (28/36; 77.8%) (Figure 3B, lower panel). The only statistically significant association between phylogroup and clinical variables was an inverse relationship between phylogroup B2 and recurrent UTI: only 3/36 (8.3%) of B2 isolates originated from patients with recurrent infection, compared with 12/34 (35.3%) of non-B2 isolates (OR = 0.17; 95% CI: 0.06–0.87; *p* = 0.043 for the B2 vs. non-B2 pairwise comparison; overall *p* = 0.022 across phylogroups, Table 4). No statistically significant associations were identified between individual *bla* gene carriage and clinical variables, susceptibility patterns, or biofilm formation. Regarding phylogenetic distribution, all three *bla*TEM/*bla*CTX-M co-carriage events occurred exclusively in phylogroup C isolates (3/3), whereas *bla*TEM/*bla*SHV co-carriage events were distributed across phylogroups B1, C, and A (one each), suggesting a potential role of the C lineage in the co-dissemination of these determinants; however, given the small number of doublet events, this observation should be considered exploratory. The inverse association between phylogroup B2 and recurrent UTI was maintained in adjusted logistic regression analysis after controlling for age and renal transplant history (adjusted OR = 0.19; 95% CI: 0.05–0.81; *p* = 0.025; Supplementary Table S2), confirming the robustness of this finding independently of these potential confounders. The EPV ratio for this model was 11.3, meeting the minimum threshold of ≥10 proposed by Peduzzi et al. [16].

Pairwise comparisons of antimicrobial resistance rates between each phylogroup and all remaining isolates across all 14 antimicrobial agents (Figure 3C), applying BH-FDR correction for 14 simultaneous comparisons, identified a single significant association: phylogroup B2 isolates showed significantly lower non-susceptibility to trimethoprim-sulfamethoxazole compared with non-B2 isolates (q = 0.047 for both strict R and NS definitions; Supplementary Table S3). No other phylogroup-specific resistance associations survived multiple comparison correction. Specifically, the observed associations between phylogroup C and resistance to nitrofurantoin (*p* = 0.017), TMP-SMX (*p* = 0.017), and fosfomycin (*p* = 0.008) did not remain significant after BH-FDR adjustment (q > 0.05 for all) (Supplementary Table S4).

## 3. Discussion

This study provides one of the few integrative characterizations of antimicrobial resistance, ESBL genotype, biofilm formation, and phylogenetics in *E. coli* isolated from patients admitted through the emergency department with community-onset urinary tract infections (UTIs) of sufficient severity to require inpatient management. This clinical scenario of community-onset infections selected by disease severity for hospitalization represents an epidemiologically critical interface between ambulatory and hospital medicine, where resistance patterns from the community reservoir are directly exposed and amplified. The demographic profile observed mean age 53.3 years, one-third male patients, and 18.6% renal transplant recipients reflects the high-complexity referral nature of this tertiary center and diverges substantially from the predominantly young female populations described in community-based outpatient series [17,18].

The 57.1% ESBL prevalence is particularly notable given that all isolates originated from community-onset infections. This figure substantially exceeds the ~39% ESBL rate reported for Mexican UPEC in the most comprehensive national meta-analysis to date [7] and surpasses estimates from other Latin American community-onset UTI cohorts, including 11.5% in Argentina [19], 42% among hospitalized patients in Honduras [20], and 59% in a tertiary center in Sinaloa [21]. Crucially, detecting ESBL-producing strains in community-onset infections implies that the local community reservoir is already deeply colonized by resistant organisms, independent of prior healthcare exposure, a finding with direct public health implications for empirical treatment in ambulatory and emergency settings. The absence of any statistically significant association between ESBL production and clinical host factors (age, sex, renal transplant status, or diabetes mellitus) in adjusted logistic regression analysis (Supplementary Table S2) is consistent with a community-level ecological model of ESBL dissemination rather than host-selective acquisition. In this framework, ESBL-producing *E. coli* colonize the intestinal microbiota of community-dwelling individuals independently of their comorbidity profile, and patients presenting with ESBL-producing UTI primarily reflect the local prevalence of ESBL carriage in the community reservoir from which they were colonized. This interpretation is supported by three independent systematic reviews and meta-analyses documenting a global rise in fecal carriage of ESBL-producing *E. coli* among healthy community-dwelling adults: pooled carriage rates have risen from approximately 2.6% in 2003–2005 to 21.1% by 2015–2018 [22], with community carriage now approaching rates previously considered characteristic of healthcare settings [23]. Among healthy adults without comorbidities, pooled global carriage reached 14% (95% CI: 9–20%), with ecological determinants—including antimicrobial use, international travel, and food-animal exposure—accounting for the principal variance [24]. Taken together, these data argue that the ESBL burden observed in this community-onset hospitalized cohort is a downstream consequence of community reservoir saturation rather than of patient-level risk factors, and that comorbidity-based risk stratification alone is insufficient to guide empirical antibiotic selection in high-prevalence settings. Ciprofloxacin resistance (78.6%) falls within the 55.5–85.5% range documented for UPEC in developing-country settings [25], effectively precluding empirical use in this population. After BH-FDR correction, ESBL production was significantly associated with amikacin (OR = 5.55; q = 0.002) and TMP-SMX (OR = 3.00; q = 0.036) non-susceptibility, consistent with co-localization of ESBL and non–β-lactam resistance determinants on shared mobile genetic elements [26,27,28]. Complete meropenem and cefoxitin susceptibility preserved carbapenems as the only reliably active systemic option.

In contrast to ESBL production, MDR phenotype showed significant independent associations with clinical host factors in adjusted analysis: renal transplant history (adjusted OR = 6.29; 95% CI: 1.12–35.28; *p* = 0.037) and type 2 diabetes mellitus (adjusted OR = 4.06; 95% CI: 1.15–14.40; *p* = 0.030) were both independently associated with MDR status after controlling for age and sex (Supplementary Table S5). These findings are consistent with previously reported multivariate evidence from community-acquired UTI cohorts and transplant populations. In a multicenter cohort of 988 solid-organ transplant recipients, corticosteroid-containing immunosuppression was independently associated with ESBL-Enterobacterales bloodstream infection (aOR = 1.30; 95% CI: 1.03–1.65) [29], while a Swiss nationwide prospective cohort confirmed the urinary tract as the dominant source (75%) of ESBL-Enterobacterales infection in transplant recipients, with prior antibiotic treatment as the principal independent predictor (aOR = 2.6; 95% CI: 1.0–6.8) [30]. In kidney transplant outpatients specifically, diabetes was identified as an independent risk factor for ESBL-UTI alongside prior antibiotic exposure and recurrent UTI history [31]. For type 2 diabetes, a multivariate study of community-acquired UTI (*n* = 770) identified diabetes as an independent predictor of MDR Enterobacteriaceae (aOR = 1.63; 95% CI: 1.17–2.30; *p* = 0.002) [32]; a geographically relevant Mexican cohort from the same region further reported that diabetes was significantly associated with ESBL-producing uropathogens (OR = 2.8; 95% CI: 1.2–6.7) [33], reinforcing the regional validity of this association. The mechanistic substrate differs between the two comorbidities: in transplant recipients, calcineurin inhibitor-driven impairment of cell-mediated immunity and the cumulative antibiotic exposure associated with post-transplant prophylactic and therapeutic protocols generate sustained selective pressure favoring MDR strains [29,30]; in diabetic patients, compromised innate urinary tract immunity (including impaired neutrophil function, reduced uroepithelial defensin secretion, and glycosuria-facilitated bacterial growth) creates a permissive environment for colonization and persistence of antibiotic-resistant organisms [32,33]. Notably, the borderline associations observed for renal transplant (aOR = 4.35; *p* = 0.063) and diabetes (aOR = 3.44; *p* = 0.053) in the ESBL model suggest that these comorbidities may influence resistance acquisition through pathways related to total antibiotic exposure rather than ESBL-selective pressure specifically; the limited sample size precludes definitive conclusions and prospective studies are required.

The predominance of *bla*TEM among ESBL producers (85.0%) constitutes the most distinctive finding of this study and departs markedly from national and global ESBL epidemiology. In Mexico, CTX-M-1 group enzymes dominate national surveillance (55.6%) [7], and recent multicenter genotyping data confirm CTX-M-1-8 and CTX-M-9 as principal regional variants [21,34]; CTX-M is similarly dominant in other high-resistance settings, where *bla*TEM occurs only as a co-carriage gene [35]. The fact that this pattern emerged exclusively from community-onset isolates reinforces two non-exclusive mechanistic explanations. First, the sustained community-level use of aminopenicillins and early-generation cephalosporins may maintain high-prevalence *bla*TEM-1 reservoirs in the local microbiota, providing the mutational substrate from which TEM-type ESBL variants emerge under antibiotic selection—consistent with the collateral expansion framework [36] and with the well-established dependence of TEM-derived ESBL evolution on prior TEM-1 background prevalence [37]. This dynamic operates at the community level without requiring healthcare exposure, explaining why it manifests in patients presenting de novo from the community. Second, reduced local circulation of ST131-H30Rx—the principal global vector of CTX-M-15 [38]—may limit the competitive advantage of CTX-M-carrying lineages locally. Consistent with established evidence, no associations were detected between ESBL genotype and resistance phenotype, clinical severity, or phylogroup [39,40].

Biofilm formation (77.1%) was consistent with global UPEC estimates [9] and contemporaneous Mexican [8], Argentinian [19], and Egyptian [41] data. No significant associations were identified between biofilm intensity and ESBL/MDR phenotypes, resistance genes, or clinical outcomes, corroborating the mechanistic independence of biofilm from horizontally acquired resistance [42,43]: chromosomally encoded UPEC biofilm pathways (FimH, curli, c-di-GMP) are structurally distinct from plasmid-borne ESBL and aminoglycoside resistance mechanisms [42]. This independence is further supported by an inverse biofilm-resistance relationship in Egyptian UPEC [44] and by genomic evidence localizing biofilm-associated and resistance genes to separate islands within the same clinical strains [45].

Phylogroup B2 predominated (51.4%), consistent with national and global UPEC data [7,10,19,20,46,47,48,49]. The inverse association between phylogroup B2 and recurrent UTI was maintained after adjustment for age and renal transplant history (adjusted OR = 0.19; 95% CI: 0.05–0.81; *p* = 0.025; Supplementary Table S6), reflecting two converging mechanisms. First, prior antibiotic exposure preferentially depletes B2 from the infecting pool: susceptible B2 strains are eliminated by treatment while more resistant non-B2 phylogroups (A, D, C) are selectively enriched [50]; this occurs because B2 strains acquiring quinolone or TMP-SMX resistance lose key urovirulence determinants—including hemolysin, CNF-1, and S/F1C fimbriae—at a fitness cost that reduces their competitiveness in the urinary niche [51,52]. Second, B2’s extensive virulence arsenal drives acute symptomatic presentations that prompt earlier treatment, limiting persistence and thereby reducing recurrence probability [48]. Although some studies report continued B2 dominance in relapsing infections attributable to intracellular bacterial community formation [53]. The sole association surviving BH-FDR correction (lower TMP-SMX non-susceptibility in B2 (q = 0.047)), is attributable to fitness costs imposed by co-maintaining large chromosomal virulence islands alongside plasmid-borne resistance determinants [51,52,54]. Phylogroup C exhibited the highest MDR prevalence (81.3%) and one of the highest biofilm formation rates (87.5%) in our collection, consistent with independent clinical evidence: adhesin genes and antimicrobial resistance were disproportionately concentrated in phylogroups B2 and C among hospitalized UPEC in Thailand [55], and a WGS surveillance study identified phylogroup C (predominantly ST410, co-carrying *bla*NDM-5 and *bla*CTX-M-15) as the dominant lineage (54.4%) among carbapenem-resistant urinary *E. coli* [56]. Its high prevalence in this UPEC collection warrants a clarification regarding pathotype identity: although phylogroup C is classically associated with EHEC strains carrying *stx1*, *stx2*, and *eae*, the Clermont quadruplex PCR constitutes a phylogenetic classification based on chromosomal structural markers, not a pathotype designation; all 70 isolates in our study were recovered from urine cultures of patients with clinically confirmed UTI (a presentation incompatible with the enteric EHEC pathotype). The rising recognition of non-EHEC phylogroup C lineages in extra-intestinal infections likely reflects clonal expansion of MDR ExPEC within the C clade, driven by plasmid-mediated resistance acquisition [10,56]. Virulence gene characterization (*stx1*, *stx2*, *eae*, *fimH*, *hlyA*) was not performed, representing a prioritized objective for future work. Although C-specific associations did not survive BH-FDR correction, the convergent evidence of high MDR and biofilm prevalence across independent cohorts [10,20,55], identifies this lineage as a candidate for prospective regional surveillance.

The resulting therapeutic landscape is critically constrained. Co-resistance to fluoroquinolones and TMP-SMX in ESBL producers eliminates the principal oral agents for pyelonephritis management. Although fosfomycin (4.3% NS) and nitrofurantoin (11.4% NS) retained favorable in vitro profiles, current IDSA guidance restricts both to uncomplicated lower UTI only, as neither achieves adequate parenchymal or systemic concentrations for upper tract or bacteremic disease [57], directly relevant given that 51.4% of patients presented with pyelonephritis and 24.3% with urosepsis. The FOREST trial confirmed IV fosfomycin did not meet noninferiority criteria for MDR bacteremic UTI [58]. Real-world data from the same city document carbapenem use in >75% of first UTI episodes in kidney transplant recipients [59], consistent with the complete meropenem susceptibility observed here. This is especially relevant given the 18.6% transplant prevalence: MDR colonization increases one-year mortality 2.35-fold in solid organ transplant recipients [60], and ESBL-UTI is associated with significantly higher hospitalization and recurrence rates in this population [61]. The 72% meropenem resistance reported in an Iranian transplant cohort [62] underscores the critical importance of stewardship measures to preserve carbapenem activity locally. It must be emphasized that these therapeutic implications are based exclusively on in vitro susceptibility data interpreted in the context of external clinical guidelines; this study did not prospectively capture treatment regimens, therapeutic responses, or patient outcomes attributable to specific antimicrobial agents. These recommendations should therefore be understood as microbiologically informed guidance requiring validation in prospective pharmacokinetic-pharmacodynamic and clinical outcome studies.

Limitations include the single-center design and moderate sample size (*N* = 70), which reduces power for subgroup analyses and limits generalizability. No a priori sample size calculation was performed, as the study enrolled all eligible consecutive isolates over a fixed 12-month surveillance period. With *N* = 70 isolates and a markedly unequal phylogroup distribution, the study has limited statistical power for pairwise phylogroup comparisons: phylogroups D (*n* = 4), B1 (*n* = 3), and E (*n* = 1) are substantially underpowered, and only associations of large magnitude (relative risk ≥ 2.5 or greater) would be detectable at 80% power even at the full collection level. All comparisons involving minor phylogroups must therefore be interpreted as purely descriptive and hypothesis-generating. Retrospective data collection introduced potential selection bias and incomplete clinical information. Molecular characterization relied on targeted PCR without whole-genome sequencing or MLST, precluding clonal analysis, plasmid replicon characterization, and mobile genetic element mapping [45]. Furthermore, virulence gene profiling was not performed; characterization of urovirulence determinants, particularly type 1 fimbriae (*fimH*), P fimbriae (*papG*), α-hemolysin (*hlyA*), cytotoxic necrotizing factor (*cnf-1*), and markers associated with intracellular bacterial community (IBC) formation, would be essential to mechanistically explain the unexpected prevalence and clinical pathogenicity of phylogroup C isolates, and to fully interpret the inverse association between phylogroup B2 and recurrent UTI. Virulence gene characterization, ideally integrated with whole-genome sequencing, represents a prioritized objective for future studies in this clinical setting. Longitudinal follow-up was unavailable for clinical outcome assessment beyond the index hospitalization.

## 4. Materials and Methods

### 4.1. Isolate Selection and Species Confirmation

This study was designed as a single-center retrospective observational study (analytical cross-sectional design) and was conducted in accordance with the ethical principles outlined in the Declaration of Helsinki. The study was carried out at the Hospital Civil de Guadalajara ‘Fray Antonio Alcalde’ (HCFAA), a tertiary-care public referral center affiliated with the Universidad de Guadalajara, serving as the primary Emergency Department referral hospital for western Mexico. Bacterial isolates and clinical records were collected consecutively over a 12-month period (September 2023–September 2024) from patients admitted through the Emergency Department. The study was approved by the Medical Ethics Committee of the Hospital Civil “Fray Antonio Alcalde” (Approval No. 137/24). The study was based on retrospective analysis of microbiological isolates and clinical records obtained during routine care, without any intervention or direct contact with patients by the research team. Patient consent was waived due to the retrospective nature of the study and the use of anonymized microbiological and clinical data, as approved by the same ethics committee.

An isolate collection was conducted at the Microbiology Laboratory of the Hospital Civil Fray Antonio Alcalde (HCFAA) using the laboratory database of all *Escherichia coli*–positive cultures obtained from hospitalized patients with urinary tract infections between September 2023 and September 2024. Inclusion criteria required that infections were community-onset, defined as UTIs presenting prior to or within 48 h of hospital admission, with no evidence of prior hospitalization or healthcare exposure within the preceding 90 days as a plausible source of infection. Samples from pregnant individuals and children were excluded from the study. The final study sample comprised 70 bacterial isolates. All isolates were recovered from urine cultures collected as part of routine diagnostic workup during the study period. Samples were obtained as midstream clean-catch urine specimens from ambulatory patients or, in patients with indwelling urinary catheters or nephrostomy tubes, via the corresponding drainage device. The microbiological criterion for significant bacteriuria was defined as ≥10^5^ CFU/mL for spontaneously voided specimens and ≥10^3^ CFU/mL for catheter-derived or otherwise instrumented samples, in accordance with CLSI M100 (35th edition) and EAU Urological Infections Guidelines (2024). Cultures yielding two or more morphologically distinct bacterial species on primary isolation (polymicrobial cultures) were classified as potentially contaminated and excluded. On the basis of sample source (urine), significant bacteriuria thresholds, and clinical presentation consistent with UTI, all 70 isolates were classified as uropathogenic *Escherichia coli* (UPEC). The unit of analysis was the isolate, with a strict criterion of one *E. coli* isolate per patient per hospitalization episode: where the laboratory database recorded more than one *E. coli*-positive urine culture from the same patient during the same admission, only the index isolate (defined as the first culture meeting microbiological inclusion criteria) was retained. No patient contributed more than one isolate to the study.

Isolates were reactivated from the microbiology laboratory biobank and re-identified using matrix-assisted laser desorption/ionization time-of-flight mass spectrometry (MALDI-TOF MS; VITEK^®^ MS, bioMérieux, Durham, NC, USA). The resulting spectra were automatically compared with a validated reference database, and species identification was accepted when the confidence score exceeded 99%.

#### Clinical Definitions

Clinical variables were operationally defined according to internationally recognized criteria to ensure reproducibility. Recurrent UTI was defined as ≥2 symptomatic episodes in the preceding 6 months or ≥3 symptomatic episodes in the preceding 12 months, per the European Association of Urology (EAU) Urological Infections Guidelines (2024) [63]. Urosepsis was defined as sepsis arising from a urinary tract source, based on Sepsis-3 consensus criteria: life-threatening organ dysfunction (Sequential Organ Failure Assessment [SOFA] score increase of ≥2 points above baseline) attributed to a dysregulated host response to a suspected or confirmed urinary infection. Septic shock was defined as a subset of urosepsis in which circulatory and cellular metabolic abnormalities are profound enough to substantially increase mortality, operationally identified by vasopressor requirement to maintain mean arterial pressure ≥ 65 mmHg with serum lactate >2 mmol/L despite adequate volume resuscitation. Pyelonephritis was diagnosed on clinical grounds: fever ≥ 38 °C, costovertebral angle tenderness, and positive urine culture, with or without imaging confirmation. Emphysematous pyelonephritis was defined as gas formation within or immediately surrounding the renal parenchyma, confirmed by computed tomography (CT). Renal abscess was defined as a focal purulent collection within the renal parenchyma confirmed by CT or ultrasound imaging.

### 4.2. Antimicrobial Susceptibility Testing

Antimicrobial resistance patterns were determined by disk diffusion on Mueller–Hinton agar following the Clinical and Laboratory Standards Institute (CLSI) M100: Performance Standards for Antimicrobial Susceptibility Testing (35th edition). Each *E. coli* isolate was tested for susceptibility to amoxicillin/clavulanic acid, ceftazidime, cefotaxime, cefuroxime, cefepime, amikacin, fosfomycin, cefoxitin, trimethoprim–sulfamethoxazole (TMP-SMX), piperacillin/tazobactam, ciprofloxacin, aztreonam, nitrofurantoin, and meropenem. The antibiotic panel was selected based on three criteria: (i) alignment with CLSI M100 recommended agents for *Enterobacteriaceae* causing urinary tract infections; (ii) correspondence with the routine diagnostic antibiogram used at HCFAA for clinical decision-making; and (iii) clinical relevance to empirical management of community-onset UTIs in western Mexico, informed by local and national resistance surveillance data [5,6,7]. Agents not included in the panel (including colistin, tigecycline, and intravenous fosfomycin) are not part of routine susceptibility testing at this institution and are reserved for extensively drug-resistant or nosocomial infections, phenotypes not represented in this community-onset cohort.

A 0.5 McFarland bacterial suspension (≈1–2 × 10^8^ CFU/mL) was prepared from a freshly isolated colony using a turbidimeter (BioSan DEN-1B). Using a sterile swab, the suspension was evenly inoculated on the surface of a Mueller–Hinton agar plate. Paper disks impregnated with the antibiotics listed above were applied using sterile forceps and incubated at 35 ± 2 °C for 16–18 h under aerobic conditions. Specifically, ceftazidime (30 µg) and cefotaxime (30 µg) disks were tested both alone and in combination with clavulanic acid (10 µg); a zone diameter increase of ≥5 mm in the combination disk compared to the cephalosporin-alone disk was interpreted as ESBL positive, per CLSI M100 (35th edition, 2025). Potential misclassification of AmpC-mediated cephalosporin resistance as ESBL was considered unlikely in this collection: all 70 isolates showed complete susceptibility to cefoxitin (0% resistance), a cephamycin that reliably serves as a surrogate marker for AmpC activity. Clinically significant AmpC overproduction—whether chromosomal (derepressed *ampC*) or plasmid-mediated—consistently confers cefoxitin resistance; the absence of such resistance across all isolates therefore argues strongly against AmpC-mediated false-positive ESBL classification in this series.

Zone diameters were interpreted according to CLSI breakpoints, which classify susceptibility as susceptible, intermediate, resistant, or susceptible–dose dependent, ensuring the accuracy and reproducibility of the antimicrobial susceptibility results. *E. coli* ATCC 25922 was used as the quality-control strain for susceptibility testing and *Klebsiella pneumoniae* ATCC 700603 as the positive ESBL control.

### 4.3. Phylogroup Identification

#### 4.3.1. DNA Extraction

Bacterial DNA was obtained by thermal lysis combined with a chloroform extraction step [64]. *E. coli* isolates were grown on nutrient agar (DIBICO 1020-A) for 18–24 h at 35 °C. A dense bacterial suspension was prepared in 200 µL of sterile injectable water, heated to 95 °C for 10–15 min, and rapidly cooled at 4 °C; this freeze–thaw cycle was repeated two to three times. After thermal lysis, the tube was centrifuged at 15,934× *g* for 15 min to remove cellular debris and obtain a crude DNA-containing supernatant. Without decanting, an equal volume of chloroform (200 µL) was added to the supernatant to remove protein and lipid contaminants. The mixture was vortexed briefly to facilitate phase separation and subsequently centrifuged at 15,934× *g* for 2 min at 4 °C. The upper aqueous phase containing DNA was carefully collected using a micropipette and stored at −20 °C until use. DNA concentration and purity were measured by spectrophotometry at 260 nm (Eppendorf BioPhotometer™ D30 (Eppendorf SE, Hamburg, Germany)), and integrity was checked by 1% agarose gel electrophoresis stained with GelRed™.

#### 4.3.2. Quadruplex PCR and Gel Electrophoresis

Phylogroups were determined using the Clermont quadruplex PCR method (2013) [15], which targets the genes *arpA*, *chuA*, *yjaA*, and the DNA fragment *TspE4.C2*. Each 25 µL reaction contained 12.5 µL of MyTaq™ 2× Mix (Meridian Bioscience, Cincinnati, OH, USA), gene-specific primers (Table 5), 1 µL of template DNA (~100 ng/µL), and nuclease-free water. The PCR conditions were as follows: initial denaturation at 94 °C for 4 min; 30 cycles of 94 °C for 5 s, 59 °C for 20 s, and 72 °C for 30 s; and a final extension at 72 °C for 5 min. Amplicons were resolved on 2% agarose gels (TBE 1×) stained with GelRed™ at 80 V for 100 min and visualized with a D-DiGit^®^ gel scanner (LI-COR Biosciences, Lincoln, NE, USA). A 100 bp DNA ladder (Gold Biotechnology, St. Louis, MO, USA) was used as a size marker. The positive control DNA was *E. coli* ATCC 25922 (phylogroup B2), the negative control DNA was *Klebsiella pneumoniae* ATCC 700603, and the no-template control contained only the reaction mix and primers.

#### 4.3.3. Phylogroup Assignment

Isolates were classified into phylogroups A, B1, B2, C, D, E, or F according to the presence or absence of target genes, following the decision scheme described by Clermont et al. [15]. (Table 6).

### 4.4. Detection of the Main Genes Associated with Extended-Spectrum β-Lactamase Production

For all isolates exhibiting an extended-spectrum β-lactamase (ESBL) phenotype, genomic DNA was extracted using a previously described protocol. The presence of the most frequently associated ESBL genes, *bla*SHV, *bla*TEM, and *bla*CTX-M, was determined by endpoint PCR using the primer sets listed in Table 7.

Each PCR reaction was prepared in a final volume of 25 µL, consisting of 12.5 µL of MyTaq™ Mix 2X (Meridian Bioscience, Cincinnati, OH, USA), which contains Taq DNA polymerase, dNTPs, MgCl_2_, and Tris-HCl buffer; 0.5 µL of each forward and reverse primer for CTX-M, TEM, and SHV; 1 µL of template DNA adjusted to approximately 100 ng/µL; and nuclease-free water to complete the final volume. Amplifications were performed in a miniAmp Plus thermocycler (Thermo Fisher Scientific, Waltham, MA, USA) under the following conditions: initial denaturation at 94 °C for 10 min, followed by 30 cycles of denaturation at 94 °C for 30 s, annealing at 61 °C for 30 s, and extension at 72 °C for 1 min, with a final extension step at 72 °C for 9 min before holding at 4 °C. PCR products were separated by electrophoresis on 2.5% agarose gels prepared in 1X TBE buffer and stained with GelRed™ (Biotium, Fremont, CA, USA). The gels were then poured into a 15 × 10 cm electrophoresis chamber (Labnet International, Edison, NJ, USA; Enduro model 12606) and filled with 1X TBE buffer. For each reaction, 3.3 µL of the PCR product was mixed with 0.7 µL of loading buffer (Sigma-Aldrich, St. Louis, MO, USA; Cat. No. G2526) and loaded into the gel wells, along with 4 µL of a 50 bp DNA ladder (Gold Biotechnology, St. Louis, MO, USA) as a molecular size marker. Electrophoresis was performed at 80 V for 90 min, and the amplicons were visualized using a D-DiGit^®^ digital gel scanner (LI-COR Biosciences, Lincoln, NE, USA; model 3500-00). Each PCR run included the appropriate controls. *Escherichia coli* ATCC 35218 (TEM-1), a previously characterized *E. coli* strain producing CTX-M, provided by the Microbiology Laboratory of the Universidad de Guadalajara at the Centro Universitario de Ciencias de la Salud (CUCS), and *Klebsiella pneumoniae* ATCC 700603 (SHV-18) were used as positive controls. *E. coli* ATCC 25922, a pansusceptible non-ESBL-producing strain, served as a negative control. A no-template control consisting of MyTaq™ Mix 2X, primers, and nuclease-free water without bacterial DNA was included in each run. The presence or absence of each β-lactamase gene was determined by comparing the amplified fragment sizes with the expected molecular weights: approximately 800 bp for *bla*TEM, 713 bp for *bla*SHV, and 655 bp for *bla*CTX-M genes.

### 4.5. Biofilm Production Assay

Biofilm formation by the *E. coli* isolates was evaluated using a standard method [66] adapted to 48-well microplates and quantified by crystal violet staining with spectrophotometric readings at 595 nm [8]. A single colony of each strain was grown overnight (18–24 h) in 3 mL Luria–Bertani (LB) broth (Dibico 4752). Cultures were diluted to an OD_600_ of 0.01 (~10^7^ CFU/mL) and 600 µL were inoculated into three independent wells per strain (biological triplicates), each processed as a separate experimental unit from the same standardized inoculum. Mean absorbance values from the three replicates were used for biofilm intensity classification. Wells yielding absorbance values deviating by more than two standard deviations from the mean of the remaining replicates for the same strain were flagged and the assay was repeated; no strain required exclusion on this basis. Wells containing sterile medium served as negative controls, and *E. coli* ATCC 25922 served as the positive control. The plates were incubated for 18–20 h at 37 °C, washed twice with sterile PBS, and air-dried for 15 min. Biofilms were stained with 1% crystal violet (Sealab 2517080) for 10 min and washed twice with deionized water. Crystal violet was solubilized with 33% acetic acid (Glacial 60-05) for 10 min, which yielded optimal absorbance readings consistent with the original protocol. Absorbance was measured at 595 nm using a Multiskan Skyhigh spectrophotometer (Thermo Fisher Scientific, Waltham, MA, USA). Biofilm production was classified following the Stepanović criteria [14] using the cut-off optical density (ODc), defined as the mean OD of negative controls plus three standard deviations. Strains were categorized as non-producer (OD ≤ ODc), weak producer (ODc < OD ≤ 2 × ODc), moderate producer (2 × ODc < OD ≤ 4 × ODc), or strong producer (OD > 4 × ODc).

### 4.6. Statistical Analyses

Statistical analyses were performed using SPSS software version 30 (IBM Corp., Armonk, NY, USA) and R version 4.3 (R Foundation for Statistical Computing, Vienna, Austria). Categorical variables are summarized as absolute frequencies and percentages. Proportions and their 95% confidence intervals were calculated using the exact binomial (Clopper-Pearson) method.

Antimicrobial susceptibility was analyzed under two complementary outcome definitions: Approach A considered isolates as Resistant (R) only, replicating conventional reporting; Approach B applied WHO-GLASS epidemiological criteria, defining non-susceptibility (NS) as the combination of Resistant, Intermediate, and Susceptibility Dose-Dependent (R + I + SDD) categories.

Statistical significance for non-multiplicity-corrected comparisons was set at *p* ≤ 0.05.

For the comparison of non-susceptibility rates between ESBL-producing vs. non-producing isolates and MDR vs. non-MDR isolates, Fisher’s exact test was applied. To control for multiple comparisons across 14 antimicrobial agents per exposure, *p*-values were adjusted using the Benjamini–Hochberg false discovery rate (BH-FDR) method, with a significance threshold of q < 0.05. The same BH-FDR correction was applied to pairwise comparisons of each phylogroup against all remaining isolates across 14 antimicrobial agents. Odds ratios (OR) with 95% confidence intervals were estimated using Fisher’s exact test with the mid-p correction where applicable. For the comparison of continuous variables across biofilm intensity categories (four groups), the Kruskal–Wallis non-parametric test was used; for categorical variables in the same context, Fisher’s exact test with Monte Carlo simulation was applied to handle cells with expected counts < 5.

To assess the independence of primary associations from potential confounding factors, three binary logistic regression models were constructed using the enter method. Model 1 examined ESBL production (yes/no) as the outcome, with age (continuous, per year), male sex, renal transplant history, and type 2 diabetes mellitus as covariates. Model 2 examined MDR status (yes/no) with the same covariates. Model 3 examined phylogroup B2 (yes/no) as the outcome, with recurrent UTI, age, and renal transplant history as covariates, given their established biological relevance to phylogroup distribution and UTI recurrence [37,39,40]. Model fit was assessed using the Hosmer–Lemeshow goodness-of-fit test. Multicollinearity was evaluated by the variance inflation factor (VIF), with a threshold of VIF < 5. The adequacy of events per variable (EPV) was evaluated per Peduzzi’s rule (EPV ≥ 10). Full model results, including unadjusted and adjusted odds ratios, are presented in Supplementary Tables S2, S5 and S6.

## 5. Conclusions

This study demonstrates a high prevalence of ESBL-producing and multidrug-resistant *E. coli* among patients with community-onset UTIs requiring hospitalization. ESBL production was significantly associated with amikacin and TMP-SMX non-susceptibility, while ciprofloxacin non-susceptibility was independently linked to MDR status rather than ESBL phenotype, a distinction with direct implications for empirical treatment selection. The predominance of *bla*TEM over *bla*CTX-M represents a distinctive local genotypic signature diverging from regional trends, consistent with sustained community-level aminopenicillin selective pressure. The inverse association of phylogroup B2 with recurrence and TMP-SMX resistance supports the clinical value of phylogenetic surveillance in guiding UTI management. Phylogroup C exhibited high MDR prevalence and biofilm formation, warranting prospective monitoring despite the exploratory nature of this finding. Complete carbapenem susceptibility underscores the urgency of local stewardship measures to preserve this therapeutic option. These findings should be interpreted within the constraints of a single-center retrospective design with moderate sample size, absence of whole-genome sequencing or virulence gene profiling, and no post-discharge follow-up. Accordingly, associations involving minor phylogroups and the therapeutic implications derived from in vitro susceptibility data remain hypothesis-generating. Nonetheless, the epidemiological signals identified here (*bla*TEM predominance, high MDR prevalence in phylogroup C, and the phylogroup B2–recurrence inverse association) constitute a locally relevant baseline that warrants prospective multicenter validation incorporating MLST-based clonal analysis and virulence factor characterization.

## Figures and Tables

**Table 1:** Clinical and epidemiological variables of patients hospitalized for urinary tract infection with microbiological isolation of *Escherichia coli*.

Variable	*N* = 70 ^1^
Mean Age (years)	53.3 (±19.6); range 18.0–96.0
Age by Ranges (years)	
18–45	24 (34.3)
46–65	28 (40.0)
>65	18 (25.7)
Sex	
Female	47 (67.1)
Male	23 (32.9)
Comorbidities	
Type 2 Diabetes Mellitus	21 (30.0)
HIV infection	3 (4.3)
Renal transplant history	13 (18.6)
Urinary catheter	7 (10.0)
Urinary tract obstruction	7 (10.0)
Urolithiasis	15 (21.4)
Prior antibiotic exposure (<3 months)	
Quinolones	8 (11.4)
Third-generation cephalosporins	4 (5.7)
Fosfomycin	2 (2.8)
Nitrofurantoin	1 (1.4)
Trimethoprim–Sulfamethoxazole	1 (1.4)
Clinical syndrome	
Cystitis	17 (24.3)
Pyelonephritis	36 (51.4)
Urosepsis	17 (24.3)
Recurrence	15 (21.4)
Clinical complication	
Septic shock	10 (14.2)
Emphysematous pyelonephritis	6 (8.5)
Renal abscess	3 (4.3)
Mortality	4 (5.7)

^1^ Data are presented as mean (±SD) with range for continuous variables, or as absolute frequency and percentage (*n* (%)) for categorical variables.

**Table 2:** Antimicrobial susceptibility profile of *Escherichia coli* isolates from hospitalized patients with community-onset urinary tract infections in Western Mexico (*N* = 70).

Antibiotic	*N*	S, *n* (%)	I, *n* (%)	SDD, *n* (%)	R, *n* (%) [95% CI]	Non-Susceptible, *n* (%) [95% CI]
Amoxicillin-clavulanate	70	36 (51.4)	15 (21.4)	0 (0.0)	19 (27.1%) [17.2–39.1]	34 (48.6%) [36.4–60.8]
Cefotaxime	70	30 (42.9)	0 (0.0)	0 (0.0)	40 (57.1%) [44.7–68.9]	40 (57.1%) [44.7–68.9]
Ceftazidime	70	31 (44.3)	0 (0.0)	0 (0.0)	39 (55.7%) [43.3–67.6]	39 (55.7%) [43.3–67.6]
Cefepime	70	33 (47.1)	0 (0.0)	13 (18.6)	24 (34.3%) [23.3–46.6]	37 (52.9%) [40.6–64.9]
Amikacin	70	35 (50)	16 (22.9)	0 (0.0)	19 (27.1%) [17.2–39.1]	35 (50%) [37.8–62.2]
Cefuroxime	70	29 (41.4)	0 (0.0)	0 (0.0)	41 (58.6%) [46.2–70.2]	41 (58.6%) [46.2–70.2]
Fosfomycin	70	67 (95.7)	0 (0.0)	0 (0.0)	3 (4.3%) [0.9–12]	3 (4.3%) [0.9–12]
Cefoxitin	70	70 (100)	0 (0.0)	0 (0.0)	0 (0%)	0 (0%)
Trimethoprim-sulfamethoxazole	70	29 (41.4)	0 (0.0)	0 (0.0)	41 (58.6%) [46.2–70.2]	41 (58.6%) [46.2–70.2]
Piperacillin-tazobactam	70	40 (57.1)	0 (0.0)	9 (12.9)	21 (30%) [19.6–42.1]	30 (42.9%) [31.1–55.3]
Ciprofloxacin	70	10 (14.3)	5 (7.1)	0 (0.0)	55 (78.6%) [67.1–87.5]	60 (85.7%) [75.3–92.9]
Aztreonam	70	31 (44.3)	0 (0.0)	0 (0.0)	39 (55.7%) [43.3–67.6]	39 (55.7%) [43.3–67.6]
Nitrofurantoin	70	62 (88.6)	2 (2.9)	0 (0.0)	6 (8.6%) [3.2–17.7]	8 (11.4%) [5.1–21.3]
Meropenem	70	70 (100)	0 (0.0)	0 (0.0)	0 (0%)	0 (0%)

Susceptibility categories were determined according to CLSI M100 breakpoints (35th edition, 2025). The Non-Susceptible (NS) category groups Intermediate (I), Susceptibility Dose-Dependent (SDD), and Resistant (R) isolates, in accordance with WHO-GLASS epidemiological surveillance criteria. 95% confidence intervals were calculated using the exact binomial (Clopper-Pearson) method. S, susceptible; I, intermediate; SDD, susceptibility dose-dependent; R, resistant; CI, confidence interval. Complete cefoxitin susceptibility across all 70 isolates (R = 0%) argues against clinically relevant AmpC overproduction as a confounding mechanism in the ESBL phenotypic confirmation.

**Table 3:** Non-susceptibility to antimicrobial agents according to ESBL-producing phenotype and multidrug resistance (MDR) status in *Escherichia coli* (*N* = 70).

Antibiotic	ESBL-Producer vs. Non-Producer (*n* = 40 vs. 30)					MDR vs. Non-MDR (*n* = 41 vs. 29)				
	ESBL+ NS, *n* (%)	ESBL− NS, *n* (%)	OR (95% CI)	*p* (Crude)	q (FDR)	MDR NS, *n* (%)	Non-MDR NS, *n* (%)	OR (95% CI)	*p* (Crude)	q (FDR)
Amoxicillin-clavulanate	31 (77.5)	3 (10.0)	28.94 (6.76–183.76)	<0.001	<0.001	30 (73.2)	4 (13.8)	16.19 (4.31–78.92)	<0.001	<0.001
Cefuroxime	40 (100.0)	1 (3.3)	NE (>91.77)	<0.001	<0.001	39 (95.1)	2 (6.9)	207.35 (28.48–3230.56)	<0.001	<0.001
Cefotaxime	40 (100.0)	0 (0.0)	NE (>153.79)	<0.001	<0.001	39 (95.1)	1 (3.4)	400.45 (39.81–16,384.00)	<0.001	<0.001
Ceftazidime	39 (97.5)	0 (0.0)	NE (>92.64)	<0.001	<0.001	38 (92.7)	1 (3.4)	281.91 (31.04–13,037.24)	<0.001	<0.001
Cefepime	37 (92.5)	0 (0.0)	NE (>51.75)	<0.001	<0.001	36 (87.8)	1 (3.4)	173.56 (21.09–7912.87)	<0.001	<0.001
Cefoxitin	0 (0.0)	0 (0.0)	—	—	—	0 (0.0)	0 (0.0)	—	—	—
Aztreonam	39 (97.5)	0 (0.0)	NE (>92.64)	<0.001	<0.001	38 (92.7)	1 (3.4)	281.91 (31.04–13,037.24)	<0.001	<0.001
Piperacillin-tazobactam	28 (70.0)	2 (6.7)	30.74 (6.20–304.89)	<0.001	<0.001	28 (68.3)	2 (6.9)	27.48 (5.58–271.53)	<0.001	<0.001
Meropenem	0 (0.0)	0 (0.0)	—	—	—	0 (0.0)	0 (0.0)	—	—	—
Ciprofloxacin	37 (92.5)	23 (76.7)	3.68 (0.75–24.28)	0.087	0.094	39 (95.1)	21 (72.4)	7.21 (1.28–75.66)	0.013	0.017
Amikacin	27 (67.5)	8 (26.7)	5.55 (1.80–18.74)	0.001	0.002	28 (68.3)	7 (24.1)	6.56 (2.07–23.24)	<0.001	<0.001
Trimethoprim-sulfamethoxazole	28 (70.0)	13 (43.3)	3.00 (1.02–9.23)	0.030	0.036	28 (68.3)	13 (44.8)	2.61 (0.89–7.96)	0.084	0.091
Fosfomycin	3 (7.5)	0 (0.0)	NE (>0.31)	0.255	0.255	3 (7.3)	0 (0.0)	NE (>0.29)	0.261	0.261
Nitrofurantoin	8 (20.0)	0 (0.0)	NE (>1.43)	0.009	0.012	8 (19.5)	0 (0.0)	NE (>1.34)	0.017	0.021

NS, non-susceptible (R + I + SDD); OR, odds ratio; 95% CI, confidence interval; q, Benjamini–Hochberg FDR-adjusted *p*-value; —, OR not estimable: outcome showed no variation across groups (100% susceptibility in both groups, i.e., cefoxitin and meropenem); NE, not estimable: near-complete or complete non-susceptibility in the exposed group precluded standard odds ratio calculation. In these cases, a one-sided 95% exact lower confidence bound is provided in parentheses. This pattern is expected for β-lactam antibiotics that are substrates of ESBL enzymes. Cefoxitin and meropenem showed 100% susceptibility across all isolates and were excluded from OR estimation. MDR, multidrug-resistant, is defined as non-susceptibility to ≥1 agent in ≥3 antimicrobial categories per Magiorakos et al. [13].

**Table 4:** Phenotypic, clinical, and genotypic characteristics by *Escherichia coli* phylogroup in hospitalized patients with community-onset urinary tract infections (*N* = 70).

Characteristic ^1^	Overall *N* = 70 ^1^	A *n* = 5 ^1^	B1 ^†^ *n* = 3 ^1^	B2 *n* = 36 ^1^	C *n* = 16 ^1^	D ^†^ *n* = 4 ^1^	E ^†^ *n* = 1 ^1^	F *n* = 5 ^1^	*p*-Value ^1^
ESBL phenotype									0.562
Non-producer	30/70 (43%)	3/5 (60%)	2/3 (67%)	16/36 (44%)	4/16 (25%)	2/4 (50%)	0/1 (0%)	3/5 (60%)	
ESBL-producer	40/70 (57%)	2/5 (40%)	1/3 (33%)	20/36 (56%)	12/16 (75%)	2/4 (50%)	1/1 (100%)	2/5 (40%)	
MDR									0.084
Non-MDR	29/70 (41%)	3/5 (60%)	3/3 (100%)	16/36 (44%)	3/16 (19%)	1/4 (25%)	0/1 (0%)	3/5 (60%)	
MDR	41/70 (59%)	2/5 (40%)	0/3 (0%)	20/36 (56%)	13/16 (81%)	3/4 (75%)	1/1 (100%)	2/5 (40%)	
Biofilm formation									0.246
Non-former	16/70 (23%)	2/5 (40%)	1/3 (33%)	8/36 (22%)	2/16 (13%)	0/4 (0%)	1/1 (100%)	2/5 (40%)	
Biofilm-former	54/70 (77%)	3/5 (60%)	2/3 (67%)	28/36 (78%)	14/16 (88%)	4/4 (100%)	0/1 (0%)	3/5 (60%)	
Biofilm intensity									0.481
Non-former	16/70 (23%)	2/5 (40%)	1/3 (33%)	8/36 (22%)	2/16 (13%)	0/4 (0%)	1/1 (100%)	2/5 (40%)	
Weak	42/70 (60%)	3/5 (60%)	1/3 (33%)	22/36 (61%)	11/16 (69%)	3/4 (75%)	0/1 (0%)	2/5 (40%)	
Moderate	8/70 (11%)	0/5 (0%)	0/3 (0%)	3/36 (8.3%)	3/16 (19%)	1/4 (25%)	0/1 (0%)	1/5 (20%)	
Strong	4/70 (5.7%)	0/5 (0%)	1/3 (33%)	3/36 (8.3%)	0/16 (0%)	0/4 (0%)	0/1 (0%)	0/5 (0%)	
*bla*TEM									0.558
Absent	36/70 (51%)	3/5 (60%)	2/3 (67%)	18/36 (50%)	6/16 (38%)	3/4 (75%)	0/1 (0%)	4/5 (80%)	
Present	34/70 (49%)	2/5 (40%)	1/3 (33%)	18/36 (50%)	10/16 (63%)	1/4 (25%)	1/1 (100%)	1/5 (20%)	
*bla*SHV									0.193
Absent	65/70 (93%)	4/5 (80%)	2/3 (67%)	35/36 (97%)	14/16 (88%)	4/4 (100%)	1/1 (100%)	5/5 (100%)	
Present	5/70 (7.1%)	1/5 (20%)	1/3 (33%)	1/36 (2.8%)	2/16 (13%)	0/4 (0%)	0/1 (0%)	0/5 (0%)	
*bla*CTX-M									0.103
Absent	63/70 (90%)	5/5 (100%)	3/3 (100%)	35/36 (97%)	12/16 (75%)	3/4 (75%)	1/1 (100%)	4/5 (80%)	
Present	7/70 (10%)	0/5 (0%)	0/3 (0%)	1/36 (2.8%)	4/16 (25%)	1/4 (25%)	0/1 (0%)	1/5 (20%)	
Clinical syndrome									0.457
Cystitis	17/70 (24%)	0/5 (0%)	1/3 (33%)	10/36 (28%)	5/16 (31%)	1/4 (25%)	0/1 (0%)	0/5 (0%)	
Pyelonephritis	36/70 (51%)	3/5 (60%)	2/3 (67%)	16/36 (44%)	7/16 (44%)	3/4 (75%)	0/1 (0%)	5/5 (100%)	
Urosepsis	17/70 (24%)	2/5 (40%)	0/3 (0%)	10/36 (28%)	4/16 (25%)	0/4 (0%)	1/1 (100%)	0/5 (0%)	
Diabetes mellitus	21/70 (30%)	1/5 (20%)	0/3 (0%)	13/36 (36%)	4/16 (25%)	1/4 (25%)	1/1 (100%)	1/5 (20%)	0.718
Recurrent UTI	15/70 (21%)	2/5 (40%)	2/3 (67%)	3/36 (8.3%)	4/16 (25%)	2/4 (50%)	0/1 (0%)	2/5 (40%)	0.022 *
Complication	19/70 (27%)	2/5 (40%)	0/3 (0%)	13/36 (36%)	3/16 (19%)	1/4 (25%)	0/1 (0%)	0/5 (0%)	0.533
Outcome									0.433
Survivor	66/70 (94%)	4/5 (80%)	3/3 (100%)	35/36 (97%)	14/16 (88%)	4/4 (100%)	1/1 (100%)	5/5 (100%)	
Death	4/70 (5.7%)	1/5 (20%)	0/3 (0%)	1/36 (2.8%)	2/16 (13%)	0/4 (0%)	0/1 (0%)	0/5 (0%)	

^1^ Data presented as *n*/*N* (%). *p*-values by Fisher’s exact test. ESBL, extended-spectrum β-lactamase; MDR, multidrug-resistant; UTI, urinary tract infection. * *p* < 0.05. ^†^ Phylogroups D (*n* = 4), B1 (*n* = 3), and E (*n* = 1): all results are purely descriptive. Statistical comparisons are not applicable due to insufficient sample size; percentages derived from these groups should be interpreted with extreme caution and no inferential conclusions should be drawn.

**Table 5:** Primers for multiplex PCR phylogenetic classification.

PCR	Gene	Sequence (5’-3’)	Bp	Reference
Quadruplex	*chuA*	F 5-ATGGTACCGGACGAACCAAC-3	288	[15]
		R 5-TGCCGCCAGTACCAAAGACA-3		
	*yjaA*	F 5-CAAACGTGAAGTGTCAGGAG-3	211	
		R 5-AATGCGTTCCTCAACCTGTG-3		
	*TspE4C2*	F 5-CACTATTCGTAAGGTCATCC-3	152	
		R 5-AGTTTATCGCTGCGGGTCGC-3		
	*arpA*	F 5-AACGCTATTCGCCAGCTTGC-3	400	
		R 5-TCTCCCCATACCGTACGCTA-3		
Group E	*arpA*	F 5-GATTCCATCTTGTCAAAATATGCC-3 R 5-GAAAAGAAAAAGAATTCCCAAGAG-3	301	
Group C	*trpA*	F-AGTTTTATGCCCAGTGCGAG-3 R-TCTGCGCCGGTCACGCCC-3	219	
Internal control (for Groups C and E)	*trpA*	F- CGGCGATAAAGACATCTTCAC-3 R- GCAACGCGGCCTGGCGGAAG-3	489	

Bp = Base pairs.

**Table 6:** Possible phylogenetic group combinations in *Escherichia coli*.

Phylogroups of *E. coli* (Clermont Classification [15])													
Gene	A	A	B1	C	E	E	E	D	D	F	B2	B2	B2
*arpA*	+	+	+	+	+	+	+	+	+	−	−	−	−
*chuA*	−	−	−	−	+	+	+	+	+	+	+	+	+
*yjaA*	−	+	−	+	−	−	+	−	−	−	+	−	+
*TspE4.C2*	−	−	+	−	−	+	−	−	+	−	−	+	+

+, presence; −, absence.

**Table 7:** Primers for the detection of β-lactamase genes in multiplex PCR.

Gene	Primer	Sequence (5′-3′)	Bp	Reference
*bla*TEM	F-TEM	5-CATTTCCGTGTCGCCCTTATTC-3	800	[65]
	R-TEM	5-CGTTCATCCATAGTTGCCTGAC-3		
*bla*SHV	F-SHV	5-AGCCGCTTGAGCAAATTAAAC-3	713	
	R-SHV	5- ATCCCGCAGATAAATCACCAC-3		
*bla*CTX-M	F-CTX-M	5-TTAGGAAGTGTGCCGCTGTA-3	655	
	R-CTX-M	5-CGGTTTTATCCCCCACAAC-3		

Bp = Base pairs.

## Data Availability

The data supporting the findings of this study are available from the corresponding author upon reasonable request.

## Supplementary Materials

The following supporting information can be downloaded at: https://www.mdpi.com/article/10.3390/antibiotics15060541/s1, Table S1: Comparison of clinical, epidemiological, and microbiological characteristics across biofilm intensity categories in *Escherichia coli* isolates recovered; Table S2. Logistic regression analysis of factors associated with ESBL-producing *Escherichia coli* among patients with community-onset urinary tract infections requiring hospitalization; Table S3: Antimicrobial resistance rates in phylogroup B2; Table S4: Antimicrobial resistance rates in phylogroup C; Table S5. Logistic regression analysis of factors associated with multidrug-resistant *Escherichia coli* among patients with community-onset urinary tract infections requiring hospitalization; Table S6. Logistic regression analysis of factors associated with phylogroup B2 *Escherichia coli* among patients with community-onset urinary tract infections requiring hospitalization.

## Abbreviations

The following abbreviations are used in this manuscript:

CFU	Colony-forming units
CI	Confidence interval
CLSI	Clinical and Laboratory Standards Institute
ESBL	Extended-spectrum β-lactamase
HCFAA	Hospital Civil Fray Antonio Alcalde
LB	Luria–Bertani
MALDI-TOF MS	Matrix-assisted laser desorption/ionization time-of-flight mass spectrometry
MDR	Multidrug-resistant
MLST	Multilocus sequence typing
ODc	Cut-off optical density
OR	Odds ratio
PBS	Phosphate-buffered saline
PCR	Polymerase chain reaction
TBE	Tris-borate-EDTA
TMP-SMX	Trimethoprim-sulfamethoxazole
UTI	Urinary tract infection
UPEC	Uropathogenic *Escherichia coli*
WGS	Whole-genome sequencing
XDR	Extensively drug-resistant
