# Supplementary material for: Antimicrobial Resistance, Biofilm Formation, and Phylogenetic Distribution of Escherichia coli in Hospitalized Patients with Community-Onset Urinary Tract Infections in Western Mexico
Source: Antibiotics (Basel). 2026 May 27;15(6):541. doi: 10.3390/antibiotics15060541 (PMC13296134; doi:10.3390/antibiotics15060541)
Supplement: Supplementary file 1 [file antibiotics-15-00541-s001.zip › Table S1. Comparison of clinical, epidemiological, and microbiological characteristics across biofilm intensity categories in Escherichia coli isolates recovered.pdf]

Table S1. Comparison of clinical, epidemiological, and microbiological characteristics across biofilm intensity categories in Escherichia coli isolates recovered

| Characteristic1 | Overall (N = 70)1 | Non-former (n = 16)1 | Weak (n = 42)1 | Moderate (n = 8)1 | Strong (n = 4)1 | p-value1 |
|---|---|---|---|---|---|---|
| Age, years | 53.3 (±19.6); range 18.0–96.0 | 61.1 (±22.6); range 22.0–96.0 | 49.9 (±17.8); range 18.0–86.0 | 51.6 (±21.8); range 20.0–82.0 | 61.8 (±15.9); range 42.0–79.0 | 0.194 |
| Sex, n (%) | | | | | | 0.616 |
| Female | 47/70 (67.1%) | 11/16 (68.8%) | 26/42 (61.9%) | 7/8 (87.5%) | 3/4 (75.0%) | |
| Male | 23/70 (32.9%) | 5/16 (31.3%) | 16/42 (38.1%) | 1/8 (12.5%) | 1/4 (25.0%) | |
| Age group, n (%) | | | | | | 0.094 |
| <45 years | 24/70 (34.3%) | 4/16 (25.0%) | 15/42 (35.7%) | 4/8 (50.0%) | 1/4 (25.0%) | |
| 46–65 years | 28/70 (40.0%) | 5/16 (31.3%) | 21/42 (50.0%) | 1/8 (12.5%) | 1/4 (25.0%) | |
| >65 years | 18/70 (25.7%) | 7/16 (43.8%) | 6/42 (14.3%) | 3/8 (37.5%) | 2/4 (50.0%) | |
| Clinical syndrome, n (%) | | | | | | 0.993 |
| Cystitis | 17/70 (24.3%) | 3/16 (18.8%) | 11/42 (26.2%) | 2/8 (25.0%) | 1/4 (25.0%) | |
| Pyelonephritis | 36/70 (51.4%) | 8/16 (50.0%) | 22/42 (52.4%) | 4/8 (50.0%) | 2/4 (50.0%) | |
| Urosepsis | 17/70 (24.3%) | 5/16 (31.3%) | 9/42 (21.4%) | 2/8 (25.0%) | 1/4 (25.0%) | |
| Recurrent UTI, n (%) | 15/70 (21.4%) | 2/16 (12.5%) | 11/42 (26.2%) | 1/8 (12.5%) | 1/4 (25.0%) | 0.685 |
| Complication, n (%) | 19/70 (27.1%) | 4/16 (25.0%) | 13/42 (31.0%) | 1/8 (12.5%) | 1/4 (25.0%) | 0.854 |
| Clinical outcome, n (%) | | | | | | 0.234 |
| Survivor | 66/70 (94.3%) | 14/16 (87.5%) | 41/42 (97.6%) | 7/8 (87.5%) | 4/4 (100.0%) | |
| Death | 4/70 (5.7%) | 2/16 (12.5%) | 1/42 (2.4%) | 1/8 (12.5%) | 0/4 (0.0%) | |
| Diabetes mellitus, n (%) | 21/70 (30.0%) | 7/16 (43.8%) | 11/42 (26.2%) | 2/8 (25.0%) | 1/4 (25.0%) | 0.602 |
| HIV infection, n (%) | 3/70 (4.3%) | 0/16 (0.0%) | 1/42 (2.4%) | 0/8 (0.0%) | 2/4 (50.0%) | 0.010 |
| Renal transplant history, n (%) | 13/70 (18.6%) | 1/16 (6.3%) | 10/42 (23.8%) | 2/8 (25.0%) | 0/4 (0.0%) | 0.351 |
| Urinary catheter, n (%) | 7/70 (10.0%) | 0/16 (0.0%) | 7/42 (16.7%) | 0/8 (0.0%) | 0/4 (0.0%) | 0.263 |
| Prior antibiotic exposure, n (%) | 14/70 (20.0%) | 4/16 (25.0%) | 9/42 (21.4%) | 0/8 (0.0%) | 1/4 (25.0%) | 0.503 |
| ESBL phenotype, n (%) | | | | | | 0.060 |
| Non-producer | 30/70 (42.9%) | 7/16 (43.8%) | 22/42 (52.4%) | 1/8 (12.5%) | 0/4 (0.0%) | |
| ESBL-producer | 40/70 (57.1%) | 9/16 (56.3%) | 20/42 (47.6%) | 7/8 (87.5%) | 4/4 (100.0%) | |
| MDR phenotype, n (%) | | | | | | 0.304 |
| Non-MDR | 29/70 (41.4%) | 7/16 (43.8%) | 20/42 (47.6%) | 1/8 (12.5%) | 1/4 (25.0%) | |
| MDR | 41/70 (58.6%) | 9/16 (56.3%) | 22/42 (52.4%) | 7/8 (87.5%) | 3/4 (75.0%) | |
| blaTEM gene, n (%) | | | | | | 0.155 |
| Absent | 36/70 (51.4%) | 9/16 (56.3%) | 24/42 (57.1%) | 3/8 (37.5%) | 0/4 (0.0%) | |
| Present | 34/70 (48.6%) | 7/16 (43.8%) | 18/42 (42.9%) | 5/8 (62.5%) | 4/4 (100.0%) | |
| blaSHV gene, n (%) | | | | | | 0.230 |
| Absent | 65/70 (92.9%) | 14/16 (87.5%) | 40/42 (95.2%) | 8/8 (100.0%) | 3/4 (75.0%) | |
| Present | 5/70 (7.1%) | 2/16 (12.5%) | 2/42 (4.8%) | 0/8 (0.0%) | 1/4 (25.0%) | |
| blaCTX-M gene, n (%) | | | | | | 0.083 |
| Absent | 63/70 (90.0%) | 15/16 (93.8%) | 39/42 (92.9%) | 5/8 (62.5%) | 4/4 (100.0%) | |

| Characteristic1 | Overall (N = 70)1 | Non-former (n = 16)1 | Weak (n = 42)1 | Moderate (n = 8)1 | Strong (n = 4)1 | p-value1 |
|---|---|---|---|---|---|---|
| Present | 7/70 (10.0%) | 1/16 (6.3%) | 3/42 (7.1%) | 3/8 (37.5%) | 0/4 (0.0%) | |

1Continuous data are presented as mean (±SD) with range. Categorical data are presented as n/N (%). Biofilm intensity was classified according to Stepanović et al. criteria: non-former (OD ≤ ODc), weak (ODc < OD ≤ 2×ODc), moderate (2×ODc < OD ≤ 4×ODc), and strong (OD > 4×ODc). p-values for continuous variables by Kruskal-Wallis test; for categorical variables by Fisher's exact test (with Monte Carlo simulation for tables with expected cell counts < 5). Bold p-values indicate p < 0.05. ESBL, extended-spectrum β-lactamase; MDR, multidrug-resistant (non-susceptibility to ≥1 agent in ≥3 antimicrobial categories); HIV, human immunodeficiency virus; UTI, urinary tract infection; OD, optical density; ODc, cut-off optical density.
