# Supplementary material for: Antimicrobial Resistance, Biofilm Formation, and Phylogenetic Distribution of Escherichia coli in Hospitalized Patients with Community-Onset Urinary Tract Infections in Western Mexico
Source: Antibiotics (Basel). 2026 May 27;15(6):541. doi: 10.3390/antibiotics15060541 (PMC13296134; doi:10.3390/antibiotics15060541)
Supplement: Supplementary file 1 [file antibiotics-15-00541-s001.zip › Table S2. Logistic regression analysis of factors associated with ESBL-producing Escherichia coli among patients with community-onset urinary tract infections requiring hospitalization.pdf]

Table S2. Logistic regression analysis of factors associated with ESBL-producing *Escherichia coli* among patients with community-onset urinary tract infections requiring hospitalization (n = 70).

Variable	Unadjusted OR (95% CI)	p-Value	Adjusted OR (95% CI) [a]	p-Value
Age (per year)	1.00 (0.97–1.02)	0.861	1.00 (0.97–1.03)	0.953
Male sex	0.57 (0.21–1.56)	0.273	0.66 (0.23–1.94)	0.450
Renal transplant	3.00 (0.75–12.05)	0.122	4.35 (0.93–20.45) †	0.063
Diabetes mellitus	2.40 (0.80–7.21)	0.119	3.44 (0.98–11.99) †	0.053

[a] Adjusted simultaneously for all variables listed in the table (binary logistic regression, enter method). Reference categories: female sex, absence of renal transplant, absence of diabetes mellitus. *p<0.05; † p < 0.10 (borderline). OR, odds ratio; CI, confidence interval; ESBL, extended-spectrum β-lactamase. Model fit: Hosmer–Lemeshow goodness-of-fit χ^2 = 4.36, df = 6, p = 0.628; maximum variance inflation factor (VIF) = 1.41; AIC = 97.47 (null AIC = 97.61).
