# Supplementary material for: Antimicrobial Resistance, Biofilm Formation, and Phylogenetic Distribution of Escherichia coli in Hospitalized Patients with Community-Onset Urinary Tract Infections in Western Mexico
Source: Antibiotics (Basel). 2026 May 27;15(6):541. doi: 10.3390/antibiotics15060541 (PMC13296134; doi:10.3390/antibiotics15060541)
Supplement: Supplementary file 1 [file antibiotics-15-00541-s001.zip › Table S3. Antimicrobial resistance rates in phylogroup B2.pdf]

Table S3. Antimicrobial resistance rates in phylogroup B2

Antibiotic	Approach A — Resistant (R) only [B2 n=36 ǀ non-B2 n=34]						Approach B — Non-Susceptible (R+I+SDD) [B2 n=36 ǀ non-B2 n=34]					
	**B2, n (%)**	**non-B2, n (%)**	**OR (95% CI)**	**p (crude)**	**q (FDR)**		**B2, n (%)**	**non-B2, n (%)**	**OR (95% CI)**	**p (crude)**	**q (FDR)**	
Amoxicillin-clavulanate	13/36 (36.1%)	6/34 (17.6%)	2.60 (0.77–9.74)	0.1092	0.328	ns	20/36 (55.6%)	14/34 (41.2%)	1.77 (0.63–5.13)	0.2440	0.732	ns
Cefuroxime	20/36 (55.6%)	21/34 (61.8%)	0.78 (0.27–2.23)	0.6346	0.976	ns	20/36 (55.6%)	21/34 (61.8%)	0.78 (0.27–2.23)	0.6346	0.952	ns
Cefotaxime	20/36 (55.6%)	20/34 (58.8%)	0.88 (0.30–2.50)	0.8133	0.976	ns	20/36 (55.6%)	20/34 (58.8%)	0.88 (0.30–2.50)	0.8133	1.000	ns
Ceftazidime	20/36 (55.6%)	19/34 (55.9%)	0.99 (0.35–2.81)	1.0000	1.000	ns	20/36 (55.6%)	19/34 (55.9%)	0.99 (0.35–2.81)	1.0000	1.000	ns
Cefepime	13/36 (36.1%)	11/34 (32.4%)	1.18 (0.39–3.58)	0.8044	0.976	ns	19/36 (52.8%)	18/34 (52.9%)	0.99 (0.35–2.81)	1.0000	1.000	ns
Cefoxitin	0/36 (0%)	0/34 (0%)	—	—	—	—	0/36 (0%)	0/34 (0%)	—	—	—	—
Aztreonam	20/36 (55.6%)	19/34 (55.9%)	0.99 (0.35–2.81)	1.0000	1.000	ns	20/36 (55.6%)	19/34 (55.9%)	0.99 (0.35–2.81)	1.0000	1.000	ns
Piperacillin-tazobactam	10/36 (27.8%)	11/34 (32.4%)	0.81 (0.25–2.53)	0.7956	0.976	ns	17/36 (47.2%)	13/34 (38.2%)	1.44 (0.50–4.18)	0.4783	0.871	ns
Meropenem	0/36 (0%)	0/34 (0%)	—	—	—	—	0/36 (0%)	0/34 (0%)	—	—	—	—

Ciprofloxacin	30/36 (83.3%)	25/34 (73.5%)	1.78 (0.49–7.00)	0.3888	0.844	ns	32/36 (88.9%)	28/34 (82.4%)	1.70 (0.36–9.07)	0.5079	0.871	ns
Amikacin	13/36 (36.1%)	6/34 (17.6%)	2.60 (0.77–9.74)	0.1092	0.328	ns	21/36 (58.3%)	14/34 (41.2%)	1.98 (0.70–5.77)	0.2316	0.732	ns
Trimethoprim-sulfamethoxazole	15/36 (41.7%)	26/34 (76.5%)	0.23 (0.07–0.69)	0.0039	0.047	*	15/36 (41.7%)	26/34 (76.5%)	0.23 (0.07–0.69)	0.0039	0.047	*
Fosfomycin	0/36 (0%)	3/34 (8.8%)	0.00 (0.00–2.24)	0.1093	0.328	ns	0/36 (0%)	3/34 (8.8%)	0.00 (0.00–2.24)	0.1093	0.656	ns
Nitrofurantoin	2/36 (5.6%)	4/34 (11.8%)	0.45 (0.04–3.37)	0.4221	0.844	ns	3/36 (8.3%)	5/34 (14.7%)	0.53 (0.08–3.01)	0.4717	0.871	ns

Antimicrobial resistance rates in phylogroup B2 (n = 36) vs. all other phylogroups (non-B2; n = 34) from Escherichia coli isolates recovered from hospitalized patients with community-onset urinary tract infections in Western Mexico (n = 70 total). Approach A: outcome = Resistant (R) only. Approach B: outcome = Non-Susceptible (NS = R + I + SDD), per WHO-GLASS criteria. OR > 1 indicates higher odds of resistance in phylogroup B2 isolates. OR by Fisher's exact test. p-values adjusted by BH-FDR across 14 simultaneous comparisons (q < 0.05). †NE, not estimable (near-complete resistance in one group). *** q<0.001; ** q<0.01; * q<0.05 (BH-FDR).
