# Supplementary material for: Antimicrobial Resistance, Biofilm Formation, and Phylogenetic Distribution of Escherichia coli in Hospitalized Patients with Community-Onset Urinary Tract Infections in Western Mexico
Source: Antibiotics (Basel). 2026 May 27;15(6):541. doi: 10.3390/antibiotics15060541 (PMC13296134; doi:10.3390/antibiotics15060541)
Supplement: Supplementary file 1 [file antibiotics-15-00541-s001.zip › Table S4. Antimicrobial resistance rates in phylogroup C.pdf]

Table S4. Antimicrobial resistance rates in phylogroup C

Antibiotic	Approach A — Resistant (R) only [B2 n=36 ǀ non-B2 n=34]						Approach B — Non-Susceptible (R+I+SDD) [B2 n=36 ǀ non-B2 n=34]					
	B2, n (%)	non-B2, n (%)	OR (95% CI)	p (crude)	q (FDR)		B2, n (%)	non-B2, n (%)	OR (95% CI)	p (crude)	q (FDR)	
Amoxicillin-clavulanate	3/16 (18.8%)	16/54 (29.6%)	0.55 (0.09 – 2.42)	0.5286	0.577	ns	7/16 (43.8%)	27/54 (50%)	0.78 (0.21 – 2.75)	0.7785	0.778	ns
Cefuroxime	12/16 (75%)	29/54 (53.7%)	2.55 (0.66 – 12.26)	0.1574	0.217	ns	12/16 (75%)	29/54 (53.7%)	2.55 (0.66 – 12.26)	0.1574	0.223	ns
Cefotaxime	12/16 (75%)	28/54 (51.9%)	2.75 (0.71 – 13.19)	0.1507	0.217	ns	12/16 (75%)	28/54 (51.9%)	2.75 (0.71 – 13.19)	0.1507	0.223	ns
Ceftazidime	12/16 (75%)	27/54 (50%)	2.95 (0.77 – 14.18)	0.0927	0.185	ns	12/16 (75%)	27/54 (50%)	2.95 (0.77 – 14.18)	0.0927	0.205	ns
Cefepime	9/16 (56.2%)	15/54 (27.8%)	3.28 (0.90 – 12.50)	0.0692	0.185	ns	11/16 (68.8%)	26/54 (48.1%)	2.34 (0.64 – 9.81)	0.1671	0.223	ns
Cefoxitin	0/16 (0%)	0/54 (0%)	—	—	—	—	0/16 (0%)	0/54 (0%)	—	—	—	—
Aztreonam	12/16 (75%)	27/54 (50%)	2.95 (0.77 – 14.18)	0.0927	0.185	ns	12/16 (75%)	27/54 (50%)	2.95 (0.77 – 14.18)	0.0927	0.205	ns
Piperacillin-tazobactam	7/16 (43.8%)	14/54 (25.9%)	2.19 (0.58 – 8.16)	0.2177	0.261	ns	9/16 (56.2%)	21/54 (38.9%)	2.00 (0.56 – 7.39)	0.2583	0.310	ns
Meropenem	0/16 (0%)	0/54 (0%)	—	—	—	—	0/16 (0%)	0/54 (0%)	—	—	—	—
Ciprofloxacin	15/16 (93.8%)	40/54 (74.1%)	5.16 (0.67 –	0.1631	0.217	ns	16/16 (100%)	44/54 (81.5%)	NE† (>0.71)	0.1023	0.205	ns

			236.30)									
Amikacin	4/16 (25%)	15/54 (27.8%)	0.87 (0.18–3.49)	1.0000	1.000	ns	7/16 (43.8%)	28/54 (51.9%)	0.73 (0.20–2.56)	0.7766	0.778	ns
Trimethoprim-sulfamethoxazole	14/16 (87.5%)	27/54 (50%)	6.83 (1.36–67.78)	0.0090	0.061	ns	14/16 (87.5%)	27/54 (50%)	6.83 (1.36–67.78)	0.0090	0.051	ns
Fosfomycin	3/16 (18.8%)	0/54 (0%)	NE† (>1.50)	0.0102	0.061	ns	3/16 (18.8%)	0/54 (0%)	NE† (>1.50)	0.0102	0.051	ns
Nitrofurantoin	4/16 (25%)	2/54 (3.7%)	8.30 (1.05–101.84)	0.0217	0.087	ns	5/16 (31.2%)	3/54 (5.6%)	7.42 (1.24–55.09)	0.0128	0.051	ns

Antimicrobial resistance rates in phylogroup C (n = 16) vs. all other phylogroups (non-C; n = 54) from Escherichia coli isolates recovered from hospitalized patients with community-onset urinary tract infections in Western Mexico (n = 70 total). Approach A: outcome = Resistant (R) only. Approach B: outcome = Non-Susceptible (NS = R + I + SDD), per WHO-GLASS criteria. OR > 1 indicates higher odds of resistance in phylogroup B2 isolates. OR by Fisher's exact test. p-values adjusted by BH-FDR across 14 simultaneous comparisons (q < 0.05). †NE, not estimable (near-complete resistance in one group). *** q<0.001; ** q<0.01; * q<0.05 (BH-FDR).
