# Supplementary material for: Antimicrobial Resistance, Biofilm Formation, and Phylogenetic Distribution of Escherichia coli in Hospitalized Patients with Community-Onset Urinary Tract Infections in Western Mexico
Source: Antibiotics (Basel). 2026 May 27;15(6):541. doi: 10.3390/antibiotics15060541 (PMC13296134; doi:10.3390/antibiotics15060541)
Supplement: Supplementary file 1 [file antibiotics-15-00541-s001.zip › Table S5. Logistic regression analysis of factors associated with multidrug-resistant Escherichia coli among patients with community-onset urinary tract infections requiring hospitalization.pdf]

Table S5. Logistic regression analysis of factors associated with multidrug-resistant Escherichia coli among patients with community-onset urinary tract infections requiring hospitalization (n = 70).

**Variable**	**Unadjusted OR (95% CI)**	**p-Value**	**Adjusted OR (95% CI)** [a]	**p-Value**
Age (per year)	0.99 (0.96–1.01)	0.395	0.99 (0.96–1.02)	0.498
Male sex	0.68 (0.25–1.85)	0.448	0.87 (0.29–2.63)	0.808
Renal transplant	**4.95 (1.00–24.34) ***	**0.049**	**6.29 (1.12–35.28) ***	**0.037**
Diabetes mellitus	**2.21 (0.74–6.65)**	**0.157**	**4.06 (1.15–14.40) ***	**0.030**

[a] Adjusted simultaneously for all variables listed in the table (binary logistic regression, enter method). Reference categories: female sex, absence of renal transplant, absence of diabetes mellitus. *$p<0.05$. Bold values indicate statistically significant adjusted associations. OR, odds ratio; CI, confidence interval; MDR, multidrug-resistant. Model fit: Hosmer–Lemeshow goodness-of-fit χ^2 = 5.67, df = 6, p = 0.462; maximum VIF = 1.38; AIC = 94.59 (null AIC = 96.97).
