# Supplementary material for: Antimicrobial Resistance, Biofilm Formation, and Phylogenetic Distribution of Escherichia coli in Hospitalized Patients with Community-Onset Urinary Tract Infections in Western Mexico
Source: Antibiotics (Basel). 2026 May 27;15(6):541. doi: 10.3390/antibiotics15060541 (PMC13296134; doi:10.3390/antibiotics15060541)
Supplement: Supplementary file 1 [file antibiotics-15-00541-s001.zip › Table S6. Logistic regression analysis of factors associated with phylogroup B2 Escherichia coli among patients with community-onset urinary tract infections requiring hospitalization.pdf]

Table S6. Logistic regression analysis of factors associated with phylogroup B2 *Escherichia coli* among patients with community-onset urinary tract infections requiring hospitalization (n = 70).

Variable	Unadjusted OR (95% CI)	p-Value	Adjusted OR (95% CI) [a]	p-Value
Recurrent UTI	**0.17 (0.04–0.66) ***	**0.011**	**0.19 (0.05–0.81) ***	**0.025**
Age (per year)	1.02 (1.00–1.05)	0.101	1.01 (0.98–1.04)	0.622
Renal transplant	0.53 (0.15–1.80)	0.305	0.70 (0.16–3.09)	0.643

[a] Adjusted simultaneously for all variables listed in the table (binary logistic regression, enter method). Recurrent UTI: ≥2 symptomatic episodes within the preceding 12 months. Reference categories: absence of recurrent UTI, absence of renal transplant. *p<0.05. Bold values indicate statistically significant adjusted associations. OR, odds ratio; CI, confidence interval; UTI, urinary tract infection. Model fit: Hosmer–Lemeshow goodness-of-fit χ^2 = 4.50, df = 6, p = 0.609; maximum VIF = 1.39; AIC = 96.17 (null AIC = 98.98). Events per variable (EPV): 34 events (non-B2) / 3 predictors = EPV of 11.3, meeting the ≥10 threshold.
